# Crimean-Congo Hemorrhagic Fever, Kazakhstan, 2009–2010

**DOI:** 10.3201/eid1804.111503

**Published:** 2012-04

**Authors:** Barbara Knust, Zhumagul B. Medetov, Kakimzhan B. Kyraubayev, Yekaterina Bumburidi, Bobbie Rae Erickson, Adam MacNeil, Stuart T. Nichol, Baurzhan S. Bayserkin, Kenes S. Ospanov

**Affiliations:** Centers for Disease Control and Prevention, Atlanta, Georgia, USA (B. Knust, Y. Bumburidi, B.R. Erickson, A. MacNeil, S.T. Nichol);; Kazakhstan Ministry of Health, Almaty, Kazakhstan (Z.B. Medetov, K.B. Kyraubayev, B.S. Bayserkin, K.S. Ospanov).

**Keywords:** Crimean-Congo hemorrhagic fever, tick-borne diseases, surveillance, Kazakhstan, viruses, zoonoses

## Abstract

We evaluated Crimean-Congo hemorrhagic fever (CCHF) surveillance data from southern Kazakhstan during 2009–2010 and found both spatial and temporal association between reported tick bites and CCHF cases. Public health measures should center on preventing tick bites, increasing awareness of CCHF signs and symptoms, and adopting hospital infection control practices.

Crimean-Congo hemorrhagic fever virus (CCHFV) is a tick-borne pathogen of the *Bunyaviridae* family (*1*). The primary modes of transmission to humans are tick bites, handling of ticks, exposure to blood or tissues of viremic livestock, and direct contact with blood and body fluids of infected persons. After a 3–7-day incubation period, sudden onset of fever, myalgia, headache, and gastrointestinal symptoms develop. Hemorrhagic signs can include petechiae; cutaneous hematomas; or bleeding from the nose, gastrointestinal tract, or urogenital tract (*2*). Among hospitalized patients, case-fatality rates range from 5% to 30% (*3**,**4*).

Ticks of the genus *Hyalomma* are the primary vectors for CCHV, and the virus is endemic throughout Africa, the Middle East, eastern Europe, and central Asia. *Hyalomma* spp. ticks are 2- or 3-host parasites, and adults feed mainly on large mammals, such as livestock. Although viremia and antibodies develop in infected livestock, no disease appears to be associated with CCHFV infection (*5*).

Crimean-Congo hemorrhagic fever (CCHF) is endemic to Kazakhstan (*6**,**7*). Most CCHF cases have been reported from Southern Kazakhstan Oblast. In 2009 and 2010, reported CCHF cases increased in Southern Kazakhstan Oblast, prompting the Kazakhstan Ministry of Health to expand surveillance and disease control activities. As a part of surveillance, a tick bite reporting system was initiated. Our objectives were to summarize CCHF surveillance data and evaluate the association between reported tick bites and CCHF in Kazakhstan.

## The Study

CCHF is a reportable disease in Kazakhstan. A suspected case was defined as fever and >1 hemorrhagic sign or thrombocytopenia. A probable case was a suspected case in a person with a known risk factor for CCHF, such as a tick bite, handling of livestock, or exposure to blood or body fluids of a CCHFV-infected patient. A confirmed case was defined as laboratory evidence of infection by IgM, IgG, or antigen-capture ELISA (VECTOR-BEST, Novosibirsk, Russia), or quantitative real-time PCR (*8*). We reviewed lists of persons with confirmed and suspected CCHF cases in Southern Kazakhstan Oblast during 2009 and 2010 and compiled summary statistics. Data regarding date of disease onset and residential location were assessed.

Data on humans and animals were collected for diagnostic and surveillance purposes and were analyzed anonymously. Permission was sought from livestock owners before tick collection. No animal sampling was done as a part of this study.

Residents of Southern Kazakhstan Oblast were instructed to go to their local health care provider if they noted a tick bite. The health care provider registered them and instructed them to monitor their temperature at home for 14 days and return if fever developed. We obtained weekly summaries of tick bites and fevers reported in Southern Kazakhstan Oblast during spring and summer 2009 and 2010. In 2010, tick bites were additionally reported by rayon (local municipality). Tick bite data were compared with CCHF cases reported by date and location. We analyzed summary statistics by using standard software (SAS Institute, Inc. Cary, NC, USA) and considered p<0.05 significant. Maps were made by using geographic information systems software (Arc-GIS, ESRI, Redlands, CA, USA).

During 1999–2010, a total of 98 probable and confirmed CCHF cases were reported; 22 resulted in death. Fewer than 10 CCHF cases were reported per year, except for 1999 (19 cases), 2009 (22 cases), and 2010 (17 cases). Epidemiologic and clinical data were reviewed for 22 probable and confirmed cases in 2009 and 17 confirmed CCHF cases in 2010, all in residents of Southern Kazakhstan Oblast. An additional 34 suspected cases were identified in 2010, but sufficient data were not available for descriptive analysis. Ages of patients with probable and confirmed cases in 2009 and confirmed cases in 2010 (total of 39 patients) ranged from 0 to 72 years; 17 (44%) were 21–40 years of age. Nosocomial transmission occurred in 5 patients in 2009 and 1 patient in 2010, accounting for 15% of the cases during 2009–2010. Livestock exposures were reported for 15 (38%), and tick exposures for 13 (33%), of the CCHF case-patients. No persons with confirmed CCHF who reported a tick bite were initially recorded in the tick bite registry. CCHF was laboratory confirmed for 14 (64%) reported cases in 2009 and for 17 (100%) in 2010. Eleven (28%) case-patients died.

**Figure 1:**
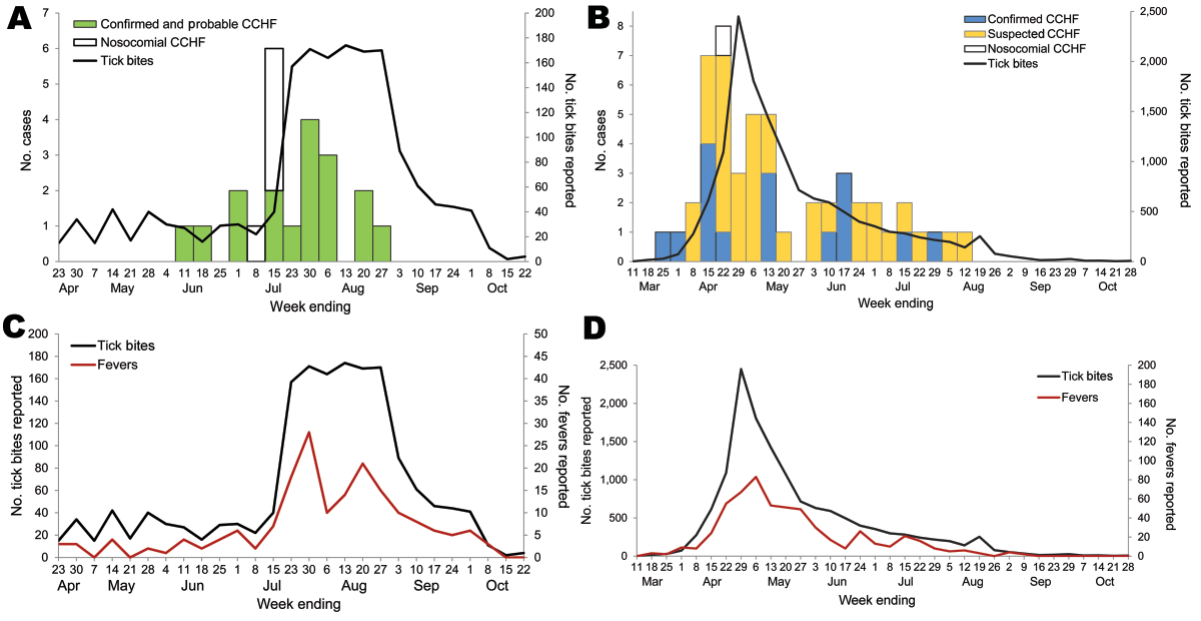
Reported Crimean-Congo hemorrhagic fever (CCHF) cases and reported tick bites in Southern Kazakhstan Oblast, Kazakhstan, April 23–October 22, 2009 (A) and March 11–October 28, 2010 (B), and reported tick bites and fevers in persons who registered a tick bite in the previous 14 days by week, April 23–October 22, 2009 (C), and March 11–October 28, 2010 (D).

Tick bite surveillance was conducted during April 17–October 22, 2009 (Figure 1, panel A) and March 3–October 28, 2010 (Figure 1, panel B). A total of 1,660 tick bites were registered in 2009; fever developed in 182 (9.7%) patients during the monitoring period (Figure 1, panel C). A total of 13,908 tick bites were registered in 2010; fever developed in 573 (4%) persons (Figure 1, panel D). In both years, peaks in reported tick bites temporally coincided with peak numbers of CCHF cases in Southern Kazakhstan Oblast; most bites and cases occurred during July–August 2009 and April–May 2010. Reported tick bites were significantly associated with number of CCHF cases per week (2009: r = 0.48, p = 0.01; 2010: r = 0.64, p<0.0001). No patients within the tick bite registry were registered as having confirmed CCHF in 2009 or 2010; however, diagnostic testing was not performed for persons who reported only a fever after a registered tick bite.

**Figure 2:**
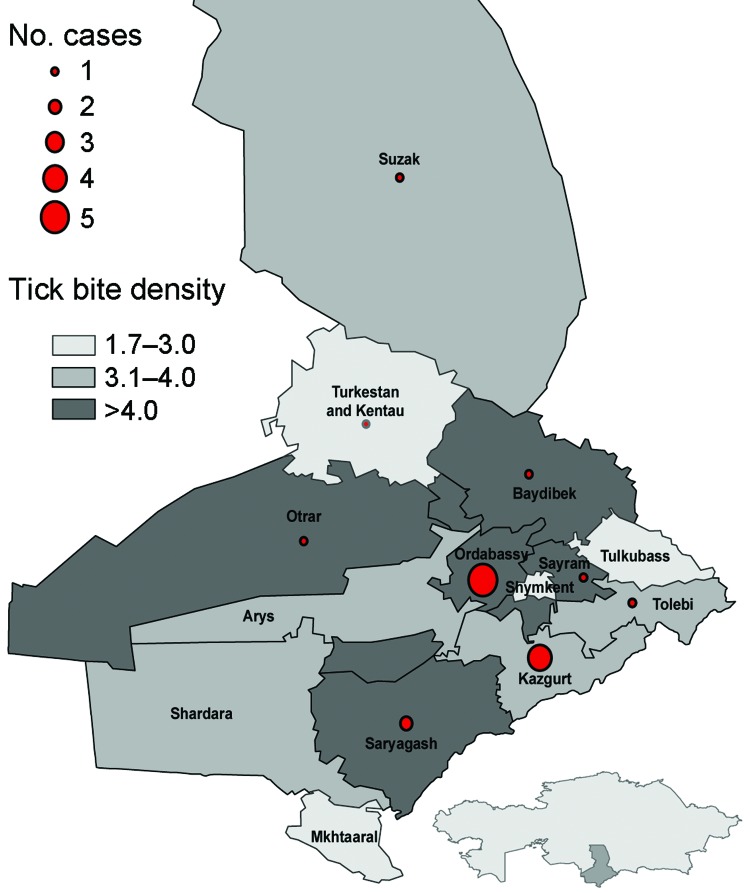
Crimean-Congo hemorrhagic fever cases and tick bite density per 1,000 persons, by rayon, Southern Kazakhstan Oblast, Kazakhstan, 2010.

For 2010, we examined the geographic distribution of reported tick bites and CCHF cases in the 15 rayons in Southern Kazakhstan Oblast (Figure 2). The tick bite density (no. tick bites registered/ 1,000 persons) varied considerably among rayons. Mean tick bite density among rayons with >1 CCHF case in 2010 (6.6 bites/1,000 persons, range 3.1–15.7) was greater than that in rayons with no CCHF cases (2.5 bites/1,000 persons, range 1.7–3.8). A nonparametric Wilcoxon rank-sum exact test found a significant difference in tick bite density scores between rayons with and without reported CCHF cases (2-sided, p = 0.01).

## Conclusions

Tick bites have long been recognized as a means of CCHFV transmission to humans (*9**–**12*), and the novel tick bite registry system provided an opportunity to examine the association between the population-level incidence of tick bites and CCHF. We demonstrated spatial and temporal correlation between reported tick bites and CCHF cases; distinct peaks in tick activity and disease were observed in both years and in regions with higher risk for CCHF in 2010. Such a registry is useful for timely deployment of tick control measures and preventive educational efforts. Exposures to ticks and livestock were commonly reported by persons with CCHF; at-risk populations should be educated about the disease and protective measures to reduce tick bites or exposure to blood and tissues of infected livestock.

Although clinical data available in this investigation were limited, we observed that disease severity of recent CCHF cases in Southern Kazakhstan Oblast are similar to those described previously in Kazakhstan and in other regions to which CCHF is endemic (*6**,**13**,**14*). The recent occurrence of nosocomial transmissions in Southern Kazakhstan Oblast underscores the need for barrier nursing techniques. Education to raise awareness among physicians of the clinical signs and symptoms, infection control measures, and treatment strategies for CCHF remains critical (*15*).

Our analysis of CCHF surveillance data in Kazakhstan found a high number of reported tick bites during the spring and summer and spatial and temporal association between tick bites and CCHF cases. Public health measures should center on preventing tick bites, increasing clinician awareness of CCHF signs and symptoms, and adopting infection control practices in hospitals.
